# Technical Features, Feasibility, and Acceptability of Augmented Telerehabilitation in Post-stroke Aphasia—Experiences From a Randomized Controlled Trial

**DOI:** 10.3389/fneur.2020.00671

**Published:** 2020-07-31

**Authors:** Hege Prag Øra, Melanie Kirmess, Marian C. Brady, Hilde Sørli, Frank Becker

**Affiliations:** ^1^Sunnaas Rehabilitation Hospital, Nesoddtangen, Norway; ^2^Institute of Clinical Medicine, University of Oslo, Oslo, Norway; ^3^Department of Special Needs Education, University of Oslo, Oslo, Norway; ^4^Nursing, Midwifery and Allied Health Professions Research Unit, Glasgow Caledonian University, Glasgow, United Kingdom

**Keywords:** aphasia, telerehabilitation, videoconference, stroke, feasibility

## Abstract

**Background:** Post-stroke aphasia is a communication disorder where existing evidence favors intensive therapy methods. Telerehabilitation represents a service model for geographically remote settings, or other barriers to clinic attendance or to facilitate an augmentation of therapy across a continuum of care. Evidence to support efficiency, feasibility, and acceptability is however still scarce. Appraising aphasia telerehabilitation in controlled trials beyond its effectiveness, by investigating feasibility and acceptability, may facilitate implementation into clinical practice.

**Methods:** In our pilot randomized controlled trial, we investigated the feasibility and acceptability of speech and language therapy by videoconference, in addition to usual care, in people with aphasia following stroke. To improve functional, expressive language, a tailored intervention was given 1 h per day, five times per week over four consecutive weeks. Feasibility measures included evaluation of technical setup using diary logs. Acceptability was investigated by examining adherence and satisfaction with therapy alongside evaluation of data safety and privacy.

**Results:** Feasibility and acceptability data were collected in relation to 556.5 h of telerehabilitation delivered to 30 participants over a 2-years intervention period by three speech-language pathologists. Protocol adherence was high, with a tolerable technical fault rate; 86 faults were registered over 541 video sessions. Most (80%; *n* = 30) of the participants experienced zero to three faults. The main cause of technical failures was flawed internet connection, causing delayed or interrupted therapy. Total satisfaction with telerehabilitation was rated good or very good by 93.1% (*n* = 29) of participants and two of three speech-language pathologists. Within a moderate variance of technical failure, participants experiencing more faults were more satisfied. No serious events regarding security and privacy were reported. Our model is feasibly and ready to be implemented across a range of clinical settings and contexts.

**Conclusions:** Synchronous telerehabilitation for post-stroke aphasia is feasible and acceptable and shows tolerable technical fault rates with high satisfaction among patients and pathologists. Within a low rate of faults, satisfaction was not negatively influenced by fault frequency. Access to clinical and technical expertise is needed when developing telerehabilitation services. Telerehabilitation may be a viable service delivery model for aphasia rehabilitation.

**Trial Registration:** ClinicalTrials.gov, ID: NCT02768922.

## Introduction

Over the last decades, services enabled by information and communications technology (ICT) have embodied a paradigm shift in the healthcare sector, where its use to increase both efficiency and accessibility of services is clearly advocated in the literature (1). In addition, the use of ICT has enabled the development of telerehabilitation, an emerging model to provide services in several disciplines of rehabilitation medicine (2, 3). In countries like Norway, with its extensive rural regions and long distances to healthcare facilities, telerehabilitation represents a flexible, low-cost, and innovative way to provide, optimize, and enable rehabilitation for different kinds of disabilities. With the use of telerehabilitation, we may provide services for those experiencing challenges in attending clinical appointments, like patients with decreased motor function and/or fatigue following stroke.

One condition that seems suitable for telerehabilitation is aphasia (4). Aphasia is a disorder seen following stroke or other causes of acquired brain injuries as a result of damage to the language-dominant hemisphere of the brain. People with aphasia may have different degrees of multimodal language impairment, like deficits in spoken language, auditory comprehension, reading, and writing. In acute stroke, aphasia is seen in a third of all cases (5) and is a predictor for outcomes in recovery (6, 7). Rehabilitation of people with aphasia is thus of importance, where existing evidence favors intensive therapy methods (8, 9).

In today's rehabilitation services, intensive aphasia rehabilitation is often not provided due to restricted resources and an uneven geographical distribution (10). In this context, speech and language therapy by videoconference represents an alternative route to make therapy more accessible in underserved and remote areas, or to accommodate the need for greater therapy dosage. In recent years, studies on aphasia telerehabilitation using both synchronously (real-time) and asynchronously (delayed) approaches have been conducted (11, 12). Customized internet videoconferencing technology offers much promise to aphasia services, with studies supporting speech and language therapy by videoconference as a viable alternative in both individual one-to-one sessions (13–15) and group-based interventions (16, 17).

Many projects involving new technology in the healthcare sector fail to reach full-scale trials or implementation into routine clinical practice (1, 18). With many promising pilot studies on aphasia telerehabilitation supporting future service delivery models, there is a need to gain more knowledge to overcome potential “pilotism.” “Pilotism” is a term used to describe how many projects involving ICT remain as projects (1), which also seems to apply to the relatively new field of aphasia telerehabilitation as most studies to date have tended to be small. We need to gather and report the feasibility of the technical features, data safety aspects, and satisfaction in larger, controlled trials to facilitate implementation into clinical services.

Existing literature within the field of telerehabilitation and telehealth highlights the importance of applying human factors in the development of telemedicine services. In creating a telerehabilitation intervention, knowledge about the characteristics of the chosen population is necessary in order to select a technology that is consistent with users' needs, skills, and contexts, thus overcoming potential barriers in using the technology (19). People with aphasia following stroke represent a heterogeneous population, where additional components like cognitive deficits, visual impairment, reduced motor function, and the presence of language impairments might interfere with their use of technology. Hence, it is vital to be able to tailor telerehabilitation services toward the targeted population of people with aphasia, exploring barriers and facilitators by including the human factor.

We investigated speech and language therapy delivered by videoconference in addition to usual care in a randomized controlled trial (RCT). The overall objective of our trial was to explore whether augmented telerehabilitation for aphasia post stroke is effective, feasible, and acceptable (20). The effect of our intervention on language outcomes has been reported elsewhere (21). The aim of this article is to describe our technical setup, including the choice of software, hardware, and our procedure for the installation of technical equipment together with user instructions. We will further present our findings in relation to feasibility and acceptability including evaluation of safety, privacy, and confidentiality. Our results consist of reports on participants' experience and data collected in relation to 556.5 h of one-to-one sessions of speech and language therapy delivered through videoconference.

## Methods and Materials

We conducted a pragmatic RCT, where augmented telerehabilitation for people with aphasia following stroke was explored (20). Participants were randomly allocated to a parallel group design to receive telerehabilitation in addition to usual care (telerehabilitation group) or to usual care alone (control group). The protocol has received ethical approval by the Norwegian Regional Committee South East for Medical and Health Research Ethics (Approval number 2015/2129) and is registered at the Clinical Trials Government (NCT02768922).

### Participants

The participants that received the telerehabilitation intervention represented a relatively unselected sample from a clinical population of people with aphasia following stroke, as broad inclusion criteria were endorsed. Participants with no limits concerning time post stroke or previous history of stroke and with Norwegian as their main language were enrolled. Participants had impairments in several language modalities, though our inclusion criteria specified naming deficits as our therapy intervention focused on spoken language. Only candidates that could not comply with the telerehabilitation intervention due to medical and/or cognitive causes were excluded.

Participants were identified and recruited from Sunnaas Rehabilitation Hospital, other rehabilitation institutions, cooperating local speech-language pathologists, and stroke units at four hospitals in the Oslo area. The research investigator (HØ) made an ambulatory visit to the participant's location for enrolment and to gain informed consent. Written informed consent was obtained from all participants and from the speech-language pathologists who delivered the telerehabilitation.

### Telerehabilitation Intervention

The Sunnaas Rehabilitation Hospital has extensive experience in the rehabilitation of patients with aphasia and the use of telemedicine and benefits from the input of a specialist telemedicine team who support the integration of telemedicine in ordinary clinical routines (22, 23). The current project was thus developed in an already well-established organizational setting with clinicians and technicians with substantial knowledge of the targeted population and wide experience from earlier and ongoing telemedicine projects. In addition, applicable components of the American Telemedicine Association's Principles for Delivering Telerehabilitation Services (24), adjusted to a Norwegian context, were integrated in our aphasia telerehabilitation project.

In our RCT, adaptations and strategies were used to increase user-friendliness and accessibility and furthermore modify the telerehabilitation to the selected patient group. The technical solution was modeled through an earlier, smaller feasibility study where personalized speech and language therapy was delivered through videoconference to four people with aphasia (25). The feasibility study identified elements in the technical arrangements requiring improvement, supporting the scaling-up of the intervention to a larger trial. Our final chosen technical setup was piloted on inpatients at Sunnaas Rehabilitation Hospital before recruitment started to our pilot trial. The telerehabilitation was delivered via videoconference from Sunnaas Rehabilitation Hospital to the participant's location (own home, institution, and rehabilitation ward). Each therapy session started with the speech-language pathologist connecting to the participant's computer by videoconference and remote-control software. After the connection was established, a “start-up” checklist (Supplementary File 1) was used at the start of each session to ensure optimal settings, privacy, and security.

The dose of the telerehabilitation intervention was 1 h per day, five times per week over four consecutive weeks. For some participants, therapy was delivered in slightly longer sessions over a smaller number of times per week, still providing the same total dosage of 20 h of telerehabilitation. The telerehabilitation was given with the intensity of 5 h per week, as this was in accordance with Norwegian national guidelines. Regarding the content of the speech and language therapy, a mixed theoretical approach was applied that included different impairment-based methods (e.g., functional-orientated and cognitive–linguistic methods). The therapy was further tailored to the participant's language impairment by both functional relevance and difficulty level, across all language modalities with a special focus on functional expressive communication. The Template for Intervention Description and Replication (TIDieR) Checklist was used to ensure transparency and replicability for future studies and to facilitate clinical implementation (20, 26).

### Hardware

Participants were provided with a portable Fujitsu PC (laptop) with necessary software and material for the intervention installed. The setup further involved a portable Jabra speakerphone to improve sound quality and a Logitech C930e webcam with a wide 90° field of view, both designed to support videoconferencing. The wide-angle web camera enabled the speech-language pathologist to see the patient's upper body, allowing the participant to use alternative communication strategies, such as body language, and gestures. A wireless computer mouse facilitated participants' control of the pointer.

The speech-language pathologist also used a portable PC, with the same installation of material and software as in the participants' computers. Each speech-language pathologist's computer was further connected to a desktop videoconference system from the Cisco TelePresence System EX Series. To establish the videoconference sessions, existing internet connection at the respective local sites was used. Various kinds of hardware were applied to access the available internet (e.g., mobile internet devices, modems, internet routers, network cables). The connection between Sunnaas Rehabilitation Hospital and the participants' computers was through Norwegian Health Net's (NHN) encrypted video service, over standard, consumer level mobile or landline broadband. The speech-language pathologists used the hospital's ordinary local network (LAN), connected via cable or over WiFi.

### Software

We used the videoconference software called Cisco Jabber/Acano from NHN. In addition, the speech-language pathologists used the software LogMeIn, which allowed them to override and remotely control the participants' computer if required. The remote-control software had [during the first feasibility study (25)] proved to be a highly valuable tool, as it supported the participants and provided assistance with computer access and technical problems. This was especially appreciated among our participants who had aphasia and, in some cases, additional cognitive impairments, apraxia, and/or limited computer skills. They only needed to turn on the computer to connect and access therapy.

The laptops were equipped with a Windows operating platform comprising Microsoft office tools. The web browser Internet Explorer was installed to access training material on the internet like maps, pictures, and easy-to-read newspapers. Lexia, a language training software, customized to facilitate retraining of language skills in people with aphasia, was also set up on all computers. The videoconference software enabled the speech-language pathologists to share presentations and material from their own computer on the screen. The LogMeIn program also allowed the speech-language pathologists to remotely select material for each session directly on the participant's computer.

### Evaluation of Security and Privacy

One of the keystones when using ICT in a healthcare services is a systematic valuation of possible threats to security and privacy, including data protection and confidentiality. In Norway, all electronic communication of personal information is regulated by national legislation, where identifiable health-related data are considered sensitive information (27). In this project, assessment of privacy and security aspects was done by a risk and vulnerability analysis (RVA) under direction of the hospital's Data Protection Office and in cooperation with the telemedicine team. The analysis was performed before the start of recruitment and under piloting of the technical setup.

The RVA indicated that there was sufficient protection of sensitive information and that the chosen technical setup adequately preserved privacy and confidentiality. The videoconference system used encrypted software and therapy sessions were live with no video recordings. Study laptops were utilized instead of participants' own computers, as LogMeIn could have enabled the speech-language pathologist to access potentially sensitive or private material. Other risk-reducing measures included completion of a “start-up” checklist at the beginning of each therapy session (Supplementary File 1). The checklist was developed as a tool to control and adjust the patient's physical environment, to optimize therapy, preserve privacy, and to confirm emergency contact details. In addition, all participants received their own user account in the videoconference software. Reuse of accounts was not endorsed. As the study laptops alternated among participants, cleaning and disinfection of the equipment using water and alcohol-based liquid or gel took place between each intervention and before delivering the equipment to the next participant. In addition, each computer was digitally cleaned and reset at the end of every intervention period to delete any used teaching material or sensitive information stored on the desktop during therapy sessions.

### Installation of Technical Equipment and User Instructions

Following baseline testing, the principal investigator (HØ) set up the equipment at the participant's location where the telerehabilitation was to take place (e.g., own home, rehabilitation ward). If possible, the speech-language pathologist who was to deliver the intervention met the participant in person before therapy started, often during baseline assessment, to support the development of a good therapeutic alliance. If this meeting could not be arranged, the speech-language pathologist and the participants met “face to face” by videoconference during the installation of the equipment at the site.

Setting up the technical equipment included connecting the participants' portable computer to the internet at the local site. This involved testing out the videoconference connection to the investigator's laptop at the site or directly to the speech-language pathologists at Sunnaas Rehabilitation Hospital, if the speech-language pathologist was not taking part in installation. After the initial installation, the internet and the participant's computer connected automatically as soon as the computer was turned on. This enhanced ease of use. Providing an internet password to attend each video session was expected to be a challenging task for most of the participants.

After connection was established, the speech-language pathologist performed a demonstration to illustrate the therapy material and videoconference software. The principal investigator (HØ), responsible for the technical installations, remained with the participant during this demonstration to address any technical difficulties and provide training. Instructions for use of the computer and software were given. A manual on how to start up the computer and begin therapy sessions was handed out alongside the “start-up” checklist (Supplementary File 1). If possible, family members and/or caregivers were also invited to take part in the demonstration and provided with user instructions.

The speech-language pathologists that delivered the therapy by videoconference received personalized training adjusted to their clinical experience, computer skills, and practice in using videoconference systems (duration of training was on average ~10 h). The training focused especially on how to use the chosen therapy materials in a telerehabilitation context, including how to use the equipment and selected software. Piloting with inpatients at Sunnaas Rehabilitation Hospital was performed in order to train the speech-language pathologists in delivering the speech and language therapy by videoconference. The technical training was given under the guidance of the telemedicine team, who also provided the necessary technical support during the intervention period. The telemedicine team consisted of both ICT personnel and clinicians with experience in the use of telerehabilitation.

### Assessments of Feasibility and Acceptability

The evaluation and assessment of the intervention's feasibility and acceptability were continuous throughout the project period. An operational definition of the two terms was used to specify which components to include in the objective measures to evaluate the telerehabilitation delivered. We defined acceptability as satisfaction with the telerehabilitation, adherence to the intervention involving withdrawal and dropout rate, and issues of privacy including safety and confidentiality. Feasibility compromised the viability of our chosen technical features like internet solution, software, hardware, and the videoconference system. The feasibility and acceptability measures are illustrated in Table 1.

**Table 1:** The feasibility and acceptability measures.

**Variable**	**Assessment tool**	**Description**
Feasibility of the technical setup	User error/Technical failure log	Number of user errors/technical problems including where the error/fault occurred and consequence of the error/fault (delayed/interrupted or canceled session)
Acceptability:		
Data safety and privacy aspects	Risk and vulnerability analysis	Location of potential risks, assessment of their potential consequences and elaboration of risk-reducing measures
Adherence to intervention	Diary log	Drop-out rate and number of sessions completed
Satisfaction with intervention	Questionnaires and semi-structured interviews	Satisfaction with intervention on a five-point scale Semi-structured interviews with speech-language pathologists and selected participants (Not included in this paper)

Our feasibility evaluation contained assessments of technical solutions where failure and technical difficulties were charted. Beyond this, feasibility measures included evaluations of the ease of use of the chosen technical solution for the participants and the speech-language pathologists. To assess feasibility of the technical setup, a log designed as a technical failure registration form was developed. The speech-language pathologist filled out this log if technical challenges arose during a videoconference session. The technical failure registration form categorized where the fault seemed to have occurred and evaluated the consequence of the given fault (Supplementary File 2). User-friendliness of the technical setup was among others assessed by labeling if the failure was a single technical problem or a user error (e.g., a participant's difficulty using the computer, software, and/or technical equipment).

Acceptability was evaluated by questionnaire where each participant and speech-language pathologist were asked to rank satisfaction on a five-point scale (Supplementary File 3). At the end of the questionnaire, each person was given the opportunity to provide general feedback in writing to further explore their experiences with the telerehabilitation. The questionnaire for the participants was modified for people with aphasia, as aphasia-accessible formatting improves comprehension of written health information (28).

In addition to the abovementioned evaluation, semi-structured interviews with selected participants and the speech-language pathologists were performed to further explore the ease of use, perception, experience, and satisfaction with the telerehabilitation intervention. These qualitative data will later be coded, analyzed, and presented in subsequent publications.

### Statistical Analysis

Statistical analysis was conducted using SPSS version 25.0 (IBM SPSS Inc., Chicago, IL, USA). Descriptive statistics were used to summarize clinical and demographic characteristics, features of the intervention, and the results of the questionnaires and technical log. In addition, descriptive statistics in the form of graphs and plots were used to explore links and relationships between various demographic variables and clinical variables toward technical feasibility and satisfaction with the therapy by videoconference. Demographic and clinical variables selected to investigate possible relationships were age, gender, auditory comprehension, and degree of disability in daily activities as measured by the modified Rankin Scale.

## Results

Feasibility and acceptability data were collected in relation to 556.5 h of speech and language therapy by videoconference delivered over a 2-years intervention period from May 2016 to June 2018. Thirty participants received speech-language telerehabilitation by videoconference in addition to usual care. The participants that received our intervention had impairments in several language modalities including naming, auditory comprehension, repetition, and the ability to produce sentences as measured by the subtest of the Norwegian Basic Aphasia Assessment (percentile score) (29) and the subtest sentence production from the Verb and Sentence Test (20 pictures with targeted sentences) (30). The Modified Rankin Scale (mRS) revealed various degrees of disability within the selected sample, where most participants were labeled as slightly or moderately disabled. The demographic and clinical characteristics of the participants including features of the telerehabilitation intervention are shown in Table 2.

**Table 2:** Demographic and clinical variables including features of the telerehabilitation.

**Variable**	**Participants who received** **telerehabilitation (*n* = 30)**
Age in years, mean (SD)	64.4 (11.7)
**Gender**, ***n*** **(%)**	
Male	19 (63.3%)
Female	11 (36.7%)
**Marital status**, ***n*** **(%)**	
Married/cohabitating	22 (73.3%)
Widow/widower	3 (10.0%)
Single	5 (16.7%)
**Housing conditions**, ***n*** **(%)**	
Independent living without home care nursing	17 (56.7%)
Independent living with home care nursing	10 (33.3%)
Sheltered housing with 24/7 care services	2 (6.7%)
Nursing home	1 (3.3%)
**Living situation** ***n*** **(%)**	
Living alone	8 (26.7%)
Living with someone	21 (70.0%)
Nursing/institution	1 (3.3%)
**Time from stroke onset in months**, ***n*** **(%)**	
≤ 3 months	14 (46.7%)
3–12 months	5 (16.7%)
≥12 months	11 (36.7%)
**Modified rankin scale at baseline**, ***n*** **(%)**	
No significant disability	-
Slight disability	14 (46.7%)
Moderate disability	8 (26.7%)
Moderately severe disability	7 (23.3%)
Severe disability	1 (3.3%)
**Language test at baseline, mean (SD)**	
NGA naming—percentile	38.6 (13.9)
NGA comprehension—percentile	47.9 (20.4)
NGA repetition—percentile	39.9 (20.5)
VAST total score	7.6 (6.2)
**Telerehabilitation intervention**	
Hours of therapy by videoconference per participant (mean)	18.6
Duration of telerehabilitation intervention in days (mean)	27.6
Total hours of SLT by videoconference delivered in the trial	556.5
Total sessions videoconference in the trial	541
**Location when receiving telerehabilitation intervention**, ***n*** **(%)**	
Own home	20 (66.7%)
Rehabilitation ward/institution	5 (16.7%)
Own home and rehabilitation ward/institution	5 (16.7%)

*NGA, Norwegian Basic Aphasia Assessment; VAST, Verb and Sentence Test, subtest sentence production; SLT, Speech-language therapy*.

## Feasibility

### Technical Failure Registration Log

There were 86 faults registered during the intervention period, occurring in 85 of the total 541 video sessions provided. The technical problems were solved by using the LogMeIn software or by giving participants and/or family members/caregivers instructions over videoconference or telephone. The primary researcher (HØ) occasionally made ambulatory visits if these initial measures failed to resolve the technical issue (~5–7 visits in total). The details of the technical failure registration log are described in Table 3.

**Table 3:** Technical failure registration log and internet solutions.

**Type of internet connection used in local settings*, n* (%)**	
Mobile 4G network	3 (10%)
Wi-Fi network in institution	3 (10%)
Broadband DSL	5 (16.7%)
Broadband Cable	7 (23.3%)
Broadband Fiber	3 (10%)
Combinations of internet (*n* = 9):	
Wi-Fi network in institution + Mobile 4G network	4 (13.3%)
Wi-Fi network in institution + Broadband Fiber	1 (3.3%)
Broadband DSL + Mobile 4G network	3 (10%)
Broadband Fiber + Mobile 4G network	1 (3.3%)
**Total number of sessions delivered by videoconference**	541
**Type of internet connection used in video sessions (% of total sessions)**	
Mobile 4G network	144 (26.6%)
Wi-Fi network in institution	99 (18.3%)
Broadband DSL	118 (21.8%)
Broadband cable	128 (23.7%)
broadband fiber	52 (9.6%)
**Type of failure**	
Technical fault	83
User error	3
Total sum of failure registered during intervention	86
**Amount of registered faults per participant, *n* (%):**	
0	7 (23%)
1–3 faults	17 (57%)
4–7 faults	3 (10%)
8 or more faults	3 (10%)
**Amount of registered faults per internet type (% of total faults)**	
Mobile 4G network	32 (37.2%)
Wi-Fi network in institution	19 (22.1%)
Broadband DSL	17 (19.8%)
Broadband Cable	13 (15.1%)
Broadband Fiber	5 (5.8%)
**Amount of sessions with faults per internet type (% of sessions per) internet type)**	
Mobile 4G network	31 (21.5%)
Wi-Fi network in institution	19 (19.2%)
Broadband DSL	17 (14.4%)
Broadband Cable	13 (10.2%)
Broadband Fiber	5 (9.6%)
**Where the faults occurred/cause of registered fault**	
SLP's computer	3
LogMeIn software	3
Unknown origin	4
Videoconference equipment	6
Videoconference software	7
Participant's computer	8
Network connection	55
**Consequence of the fault**	
Delayed training session	29
Delayed and interrupted training session	53
Canceled training session	4

Data from the log revealed a higher frequency of technical difficulties during the start of the trial, with fewer faults registered in later stages. Forty faults occurred in the first six participants, while faults registered in video sessions with the first 10 participants accounted for 70% of all failures (60 faults). The majority of the participants encountered thus a limited number of faults. Of all of the participants, seven did not have any faults registered in the technical failure registration log. Only six participants experienced more than three faults during their intervention period, where three of these six participants had more than seven faults (Table 3). The highest number of faults registered in a participant was 14.

The greatest cause for technical failures were problems with the internet connection. The log showed that there may have been an association between the type of internet service available in the local setting and the frequency of technical faults (Table 3). A Mobile 4G or Wi-Fi network within a formal institution seemed related to more technical difficulties, as 4G was used in 21.5% and Wi-Fi network in 19.2% of the sessions where faults were registered. 4G was the internet solution used most in video sessions due to its wide availability in Norway. The most frequent consequence of failures and technical difficulties, which delayed or interrupted therapy, was a reduction in quality in sound and picture due to unstable connectivity. Only 4 of the 541 video therapy sessions were canceled because of technical problems during the trial. As most of the faults were recorded as a single technical issue, the user-friendliness of the technical setup for the participants was considered adequate. When technical faults were studied using descriptive statistics regarding age, gender, auditory comprehension, and degree of disability in daily activities, no clear associations between variables were detected.

## Acceptability

### Satisfaction With the Telerehabilitation Intervention

The questionnaires return rates reporting the telerehabilitation intervention experiences were good, as only one participant failed to respond (*n* = 29). Of the participants that completed the questionnaire, 93.1% rated their overall satisfaction with therapy as good or very good. Two of the three speech-language pathologists responded in the same way. In general, participants' scores were high on satisfaction for most items. Only one participant reported the experience as “bad,” categorizing the sound and picture quality as bad. Among the speech-language pathologists, we saw a lower satisfaction rate compared to the participants as they used the response option “between good and bad” more frequently (from 33.3 to 67, 7%). One of the speech-language pathologists rated picture quality as “bad.” Results regarding satisfaction are illustrated in Table 4.

**Table 4:** Experience and satisfactory with the delivered telerehabilitation intervention, *n* (%).

**Question**	**Participants** ***n* = 29 (%)**	**Speech-language** **pathologists *n* = 3 (%)**
**1. How has it been like to receive/deliver speech-language therapy by video conference?**		
Very bad	0	0
Bad	0	0
Neither good nor bad	2 (6.9)	1 (33.3)
Good	13 (44.8)	1 (33.3)
Very good	14 (48.3)	1 (33.3)
**2. Were you satisfied with the video quality?**		
Very bad	0	0
Bad	1 (3.4)	1 (33.3)
Neither good nor bad	3 (10.3)	2 (66.7)
Good	13 (44.8)	0
Very good	12 (41.4)	0
**3. Were you satisfied with the sound quality?**		
Very bad	0	0
Bad	1 (3.4)	0
Neither good nor bad	4 (13.8)	1 (33.3)
Good	13 (44.8)	1 (33.3)
Very good	11 (37.9)	1 (33.3)
**4. Did you experience that your/the participant's language function improved by the speech-language therapy?**		
Very bad	0	0
Bad	0	0
Neither good nor bad	5 (17.2)	1 (33.3)
Good	18 (62.1)	1 (33.3)
Very good	6 (20.7)	1 (33.3)
**5. Overall, how satisfied are you with the language therapy that was received/delivered?**		
Very bad	0	0
Bad	0	0
Neither good nor bad	2 (6.9)	1 (33.3)
Good	13 (44.8)	2 (66.7)
Very good	14 (48.3)	0

In the last section of the questionnaire, the participants and the speech-language pathologists were given the opportunity to comment in general on how they experienced the received or delivered telerehabilitation. Fourteen of the participants gave feedback in their own writing or with support from family members. These comments were mainly on how the telerehabilitation intervention was perceived. Only one comment referred to technical features (which type of internet connection enabled the best sound). Feedback was also given on how the language training was regarded as good, useful/helpful, challenging, or educational. One participant reported that initially the therapy was tiring, but delivered great benefit in the end. Another participant considered the telerehabilitation received, augmenting their usual care, as a big advantage. One family member reported that the participant had become more positive and self-confident as a consequence of their participation. Involvement in the trial was described to facilitate the use of the Lexia program for self-training in addition to other therapy and greater participation in functional conversation. Several users wished to continue with speech and language therapy by videoconference after the intervention period.

The speech-language pathologists commented on how the stability in the internet connection affected the quality of the picture and sound, and how poor sound and picture quality had a negative effect on the therapy. Benefits of the delivery mode were reported, including how the therapy was time-efficient and energy-saving. One comment referred to how the intervention could be further developed, with suggestions to add utilities for training writing skills by hand. Writing was only possible by keyboard in the current setting. The wish to continue to use this form of therapy in combination with more traditional face-to-face treatment was also expressed.

**Figure 1:**
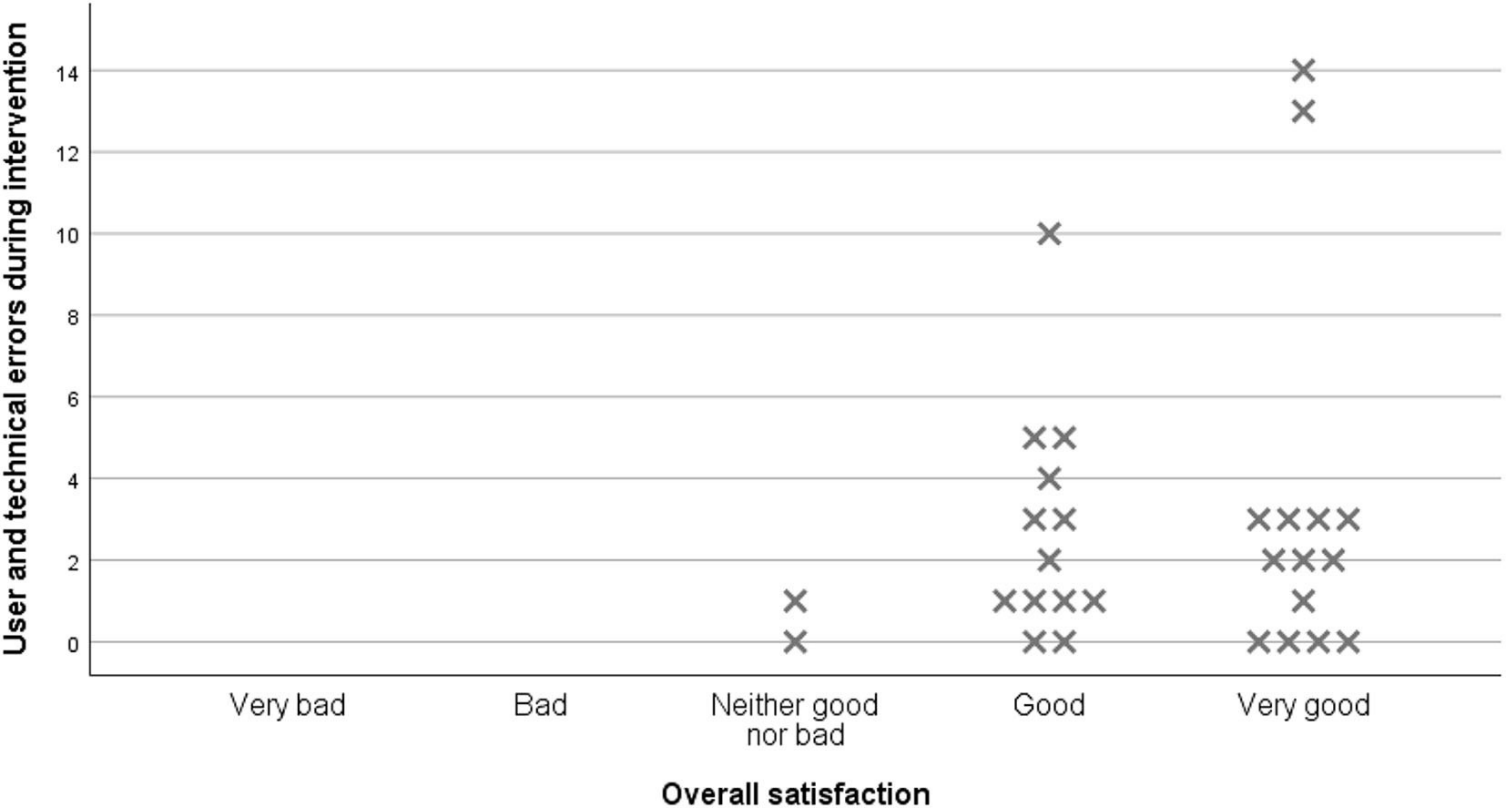
Overall satisfaction in relation to technical faults and user errors.

In summary, there seemed to be little relationship between the amount of technical failures and satisfaction with the speech and language therapy by telerehabilitation. Participants with a high frequency of technical faults still reported overall satisfaction with the intervention. The data revealed that within a moderate variance of fault rates, patients experiencing more faults were more satisfied (Figure 1). When data on satisfaction were systematically analyzed with regard to age, gender, auditory comprehension, and degree of disability in daily activities, no clear associations were detected. There might however have been a stronger connection between technical difficulties and satisfaction in the speech-language pathologists. Technical difficulties were reported by the speech-language pathologists as both challenging and frustrating, as well as having negative impact on the quality of language rehabilitation provided.

### Security, Privacy, and Adherence to the Intervention

The overall attendance at scheduled videoconference sessions was good. The protocol aimed at 20 h of speech and language therapy by videoconference over four consecutive weeks (5 h of therapy per week). To ensure a sufficient therapy time as defined per protocol, the participants were required to complete ≥16 h of speech and language therapy over 32 days. All 30 participants that received the intervention met this requirement. Most participants received speech and language therapy 60 min per day, 5 days per week over 4 weeks. In some cases, more prolonged therapy time (70–120 min per session) was given over fewer days to meet participant's broader stroke rehabilitation schedule. Hours of therapy by videoconference delivered per participant were 18.6 h (mean) over a duration of 27.6 days (mean), indicating a high acceptability and adherence to the intervention protocol. No participant withdrew during the delivery of the telerehabilitation intervention. Risk-reducing measures were successfully implemented in the protocol. Throughout the trial, no serious or adverse effects, or breaches in security, privacy, or confidentiality were reported.

## Discussion

In this study, we explored feasibility and acceptability of augmented speech and language therapy delivered by videoconference. We found high adherence to the trial protocol. All 30 participants completed the intervention per protocol requirements. There was a tolerable fault rate, as the majority of the participants experienced no or only a limited number of faults. Most faults occurred in the early stages of the trial. Technical problems caused delayed or interrupted therapy and a reduction in the quality of sound and/or picture. The main source of technical faults was the internet connection. Satisfaction with the delivered telerehabilitation was high among the participants, but somewhat lower among the speech-language pathologists. Adequate risk-reducing measures were implemented in the protocol and no serious breaches regarding security, privacy, or confidentiality were reported.

In the development of our telerehabilitation intervention, we identified several factors useful for future studies and clinical implementation of telerehabilitation. Our broad multidisciplinary team included both clinical and technical expertise and was essential to the development and delivery of a high-quality feasible technology-based intervention. In our study, the technical setup and content of the telerehabilitation intervention were developed collaboratively with ICT personnel experienced in the development and delivery of telemedicine projects. The project group also consisted of clinicians with expertise in the highly heterogeneous population of people with aphasia following stroke, which enabled important human factors to be acknowledged in our targeted sample: older people with potentially poorer digital literacy, language impairments, and possible visual and cognitive deficits. The most appropriate hardware and software for this population were identified and integrated within the technical setup, with the goal to create a technical solution that was easy to use and easy to access while preserving privacy and security. The final technical setup and intervention was the result of a long process of tailored, adaptation and piloting of an intervention clinically tested within a feasibility study prior to delivery within this larger pilot RCT. In our view, extended knowledge of both the patient group and technology was a key factor to the success of our pilot study. Dynamic development of the intervention based on multidisciplinary expertise and competencies was essential to the development of a sustainable delivery model and is an experience that future development studies and trials may draw upon.

Another strength to be highlighted is our project's pragmatic nature, which preserves the exploration of feasibility and acceptability of the intervention within local and clinical contexts. The intervention was given to a relatively unselected, heterogeneous sample of the population of people with aphasia after stroke. Earlier, we highlighted this as a challenge in evaluating the efficiency of telerehabilitation on language outcomes (21). With regards to assessment of feasibility and acceptability measures, however, we achieved a greater ecological validity by endorsing broad, clinically relevant patient participant inclusion criteria. Similarly, our telerehabilitation intervention was delivered by practicing speech and language pathologists in a clinically relevant context. Our results highlight the important benefits of adopting a pragmatic design for other studies where new rehabilitation technology is explored. Such an approach facilitates clinical implementation, as knowledge about the feasibility and acceptability of the delivery of the technology within a clinical context, among a clinically relevant population and workforce, is essential for the development of clinically useful telerehabilitation interventions.

Feasibility of our technical setup could be improved as internet instability affecting connectivity was identified as the main reason for documented technical problems. Earlier studies on aphasia telerehabilitation have identified stable bandwidth connection as imperative in ensuring that telerehabilitation services are not negatively influenced by distortions in video or audio (31). In the work by Woolf et al. (14), videoconferencing was provided by FaceTime on Macs/iPads, a videoconference software currently not permitted in healthcare services in Norway due to information security regulations. In their study, Woolf et al. reported self-ratings on the quality of technology and transmission as high. However, there appeared to be no systematic logging of technical failures, giving little indication how often picture or sound were affected by connectivity problems. In a trial by Pitt et al. (16), constraint-induced language therapy was delivered by videoconference via the Adobe connect software. In this study, the technology log revealed a number of issues with connectivity, resulting in disconnection of video and/or audio. In another study using the same setup (17), technical-related issues were reported in all treatment sessions, and in some sessions, considerable time was spent resolving technical problems. Thus, our results confirm those from earlier studies that ensuring optimal connectivity by providing sufficient internet solutions is crucial when delivering synchronous aphasia telerehabilitation. This is applicable for all forms of synchronous telerehabilitation. With regard to telerehabilitation, future technological development providing more stable and sufficient internet solutions would be especially useful. It seems to be important to document and report on technical issues in telerehabilitation research, as done in this pilot trial.

In Norway today, 9 in 10 Norwegians between 16 and 79 years use the internet on a daily basis (32). Internet usage has grown rapidly over the last decades, where we have seen an increase in number of households subscribed to broadband together with a continuous rise in median internet speed throughout the country (33). As information on the current internet market in the trial's geographical setup suggested an adequate infrastructure, and in the context of our pragmatic trial, we decided to rely on internet solutions available in the participants' local settings. Our results suggest however that the established infrastructure may not always comply with the demands needed to deliver high-quality live videoconferencing. The number of devices and people using the network simultaneously during treatment sessions (peak internet usage times), together with other factors like reduced signal in brick buildings and large distance to router, may have influenced connectivity. As our video sessions were live, demands on internet quality, capacity, and speed were higher compared to other streaming activities.

In hindsight, closer evaluation of the internet solution in each setting could have been performed before the start of therapy. Assessment of internet connection quality could have been integrated in the protocol to a greater extent to safeguard optimal connection for the videoconference. In addition, the technical log did not contain measures of median Mbit/s. Though difficult to monitor, information on median Mbit/s could have been used to map network data transfer rates vital to provide optimal video sessions.

Technical difficulties were highest in the trial start and declined during the course of the investigation. This has also been reported previously in other videoconferencing trials (34). This indicates that projects involving ICT go through a dynamic process that adds to an already complex intervention. Thus, there might be a need for an even longer piloting period than in more traditional RCTs. This should be considered when planning studies on telerehabilitation interventions. An important key factor to aid technical problems in our trial was the remote-control software. The LogMeIn software was a highly valuable tool to endorse ease of use and assist with technical challenges. We suspect that its use had a positive effect on satisfaction in both the participants and the speech-language pathologists in our study. We recommend the use of a remote-control software especially for patient groups with cognitive or communication impairments as it enhances acceptability when technical support can be provided remotely by both therapist and technicians.

High rates of satisfaction in combination with few user-related errors indicate high acceptability of our intervention. The non-physical presence of the speech-language pathologist during sessions was not explicitly examined in our questionnaire on satisfaction. This was however not a frequent topic mediated during conversation with participants, but could be interesting to further investigate in the future.

When analyzing the data, there seems to be little relation between the number of technical failures and participants' satisfaction. In general, a high degree of satisfaction with the technology was reported in the questionnaire (Table 4). This conforms with the earlier referred studies, where problems with connectivity were well-tolerated (14) and high satisfaction with technology was noted, despite problems with the transmission logged (16). In our trial, participants that experienced faults more frequently actually reported higher satisfaction levels. This might be a result of participants anticipating a level of technical difficulties and a variation of connectivity. The analysis of our qualitative data might shed further light on these results.

The speech-language pathologists' satisfaction ratings were somewhat lower as they reported poor sound and video quality, negatively affecting the quality of the training. As the SLPs provided many hours of therapy to different participants, they gained a broader picture of the delivered intervention and technical setup than the participants. This may have guided their ratings. In addition, it is also important to acknowledge that SLPs perform a number of tasks when delivering telerehabilitation as they handle technical challenges simultaneously with providing therapy. This may lead to higher requirements in the SLPs compared to the participants. There is a need to explore this further, also because the current sample is limited.

It is expected that future stroke rehabilitation services will increasingly integrate technology in therapy and training compared to today's services. There is a need for innovative thinking as an aging population, increased survival rates following stroke, and increasing fiscal constraints will demand healthcare resources beyond existing capacity in most countries. In our current trial, speech and language therapy by videoconference successfully augmented therapy time with a significant impact and effect on language outcomes for people with aphasia post stroke (21). Future trials should also investigate the possibility of telerehabilitation as a replacement to traditional face-to-face aphasia therapy, including effects on language function, patient and therapist experiences, tolerance to high-intensity interventions, as well as economical aspects. Further, comparative studies of different types of telerehabilitation (e.g., videoconferencing vs. asynchronous aphasia telerehabilitation) should be performed. While videoconferencing as a synchronous telerehabilitation method allows frequent direct contact with the speech-language pathologist as well as therapist-guided language training at home, asynchronous methods add flexibility for the patient and can further augment therapy intensity. Also, especially in the light of potential issues associated with connectivity instability, the use of hybrid approaches might be useful as, e.g., used in speech treatment for Parkinson's disease (35). Future research also needs to address whether particular telerehabilitation methods are more or less beneficial and acceptable for particular subgroups of people with aphasia, and how combinations of therapy delivery models might be optimized for the benefit of the patient.

In the new world of telerehabilitation, this study highlights the extra complexity that ICT adds to a rehabilitation intervention. An extended multidisciplinary approach where clinicians and ICT personnel work collaboratively was essentially for the development of our successful intervention and is thus recommended for future work in this field. In addition, efforts to establish optimal internet settings and solutions may inquire a greater cooperation with internet service and videoconference system providers. The demands of live videoconferencing on an internet connection are also important considerations in future trials and in the implementation of future clinical services.

Despite these challenges, our key findings suggest that telerehabilitation for aphasia may be a viable future service delivery model. Our pilot trial results suggest that our current intervention improves language functions and is acceptable to a clinically relevant patient group and therapists with high satisfaction rates. We consider this model ready to be implemented and evaluated on a larger scale and across different clinical contexts.

## Data Availability Statement

The datasets generated for this study will not be made publicly available. The dataset for this article is not publicly available because of Norwegian law regarding information privacy. Excerpts of the data can be made available on requests to the corresponding author.

## Ethics Statement

This study involving human participants was reviewed and approved by Norwegian Regional Committee South East for Medical and Health Research Ethics. The patients/participants provided their written informed consent to participate in this study.

## Author Contributions

HØ is a Ph.D. fellow and the principal investigator of the study. She contributed to the protocol design, performed statistical analysis, and drafted this manuscript. FB conceived the study and is the project manager and main supervisor for the project. MK and MB are co-supervisors. FB, MK, MB, and HS contributed to the design of the protocol, the conduction of the study, and the writing of this manuscript. All authors read and approved the final manuscript.

## Conflict of Interest

The authors declare that the research was conducted in the absence of any commercial or financial relationships that could be construed as a potential conflict of interest.
