# Supplementary material for: Technical Features, Feasibility, and Acceptability of Augmented Telerehabilitation in Post-stroke Aphasia—Experiences From a Randomized Controlled Trial
Source: Front Neurol. 2020 Jul 31;11:671. doi: 10.3389/fneur.2020.00671 (PMC7411384; doi:10.3389/fneur.2020.00671)
Supplement: Supplementary file 1 [file Data_Sheet_1.PDF]

Checklist for the speech-language pathologist before therapy

Before the videosession can start, we want you to review the following together with the participant, relatives and potential healthcare professionals present:

1. **Start with a presentation of everybody in the room on both side**

2. **Clarify security / privacy locally:**

- Is there anybody else present in the room or nearby that the camera does not capture?
- Is there visibility for others in the room or can sound be heard by others?

3. **Go through the participant's checklist:**

- Is your mobile phone turned off / on soundless mode?
- Is the TV / radio nearby turned off?
- Are you sitting well at the desk / is the table adjusted to the right height?
- Is your computer placed in the right position for you and the speech-language pathologist?
- Are those around you informed that the speech-language therapy is starting? You will be busy with this for the next hour.

4. **Run through the emergency procedure**
(from the E-Manual chapter 16 on "Videoconference Home Consultation"):

- Is there another person present in the room or close by during the therapy session? (should previously been given both oral and written information about this).
- Does the speech-language pathologist who participates in the videosession carry a cell phone/portable phone during the session?
- Does the speech-language pathologist during the therapy session have access to the mobile number of the local contact person? Information about the participant's address in the event of an emergency?

[In addition, the checklist includes phone numbers of user help services and contact persons.]
