# Supplementary material for: Technical Features, Feasibility, and Acceptability of Augmented Telerehabilitation in Post-stroke Aphasia—Experiences From a Randomized Controlled Trial
Source: Front Neurol. 2020 Jul 31;11:671. doi: 10.3389/fneur.2020.00671 (PMC7411384; doi:10.3389/fneur.2020.00671)
Supplement: Supplementary file 2 [file Data_Sheet_2.PDF]

Error registration form

Log for technical errors during the telemedical speech-language therapy

Date: ______________

Participant: __________________

How did the error occur?

Where did the error occur?

- ☐ In the speech–language pathologist´s computer
- ☐ In the participant´s computer
- ☐ In the internet connection
- ☐ In Jabber/Acano
- ☐ In Lexia
- ☐ In the remote control software (LogMeIn)
- ☐ Unknown
- ☐ Other, describe under:

Was this a technical error or a user error?

- ☐ Technical error
- ☐ User error

What was the consequence of the error?

- ☐ Delayed training session
- ☐ Interrupted training session
- ☐ Other consequences, describe below or at the back of the form:
