# Supplementary material for: Technical Features, Feasibility, and Acceptability of Augmented Telerehabilitation in Post-stroke Aphasia—Experiences From a Randomized Controlled Trial
Source: Front Neurol. 2020 Jul 31;11:671. doi: 10.3389/fneur.2020.00671 (PMC7411384; doi:10.3389/fneur.2020.00671)
Supplement: Supplementary file 3 [file Data_Sheet_3.PDF]

Questionnaire to participants

1) How has it been like to receive speech-language therapy by video conference?

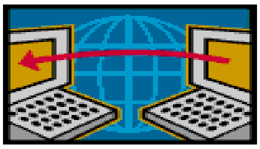

++ ☺	+	0	-	-- ☹
Very good	Good	Neither good nor bad	Bad	Very bad

2) Were you satisfied with video quality?

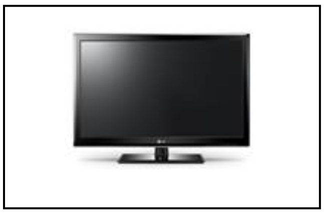

++ ☺	+	0	-	-- ☹
Very good	Good	Neither good nor bad	Bad	Very bad

3) Were you satisfied with sound quality?

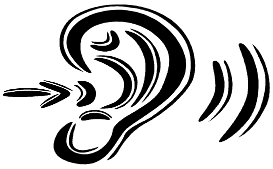

++ ☺	+	0	-	-- ☹
Very good	Good	Neither good nor bad	Bad	Very bad

4) Did you experience that your language function improved by the speech-language therapy?

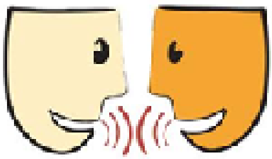

++ ☺	+	0	-	-- ☹
Very good	Good	Neither good nor bad	Bad	Very bad

5) Overall, how satisfied are you with the language therapy you received?

++ ☺	+	0	-	-- ☹
Very good	Good	Neither good nor bad	Bad	Very bad

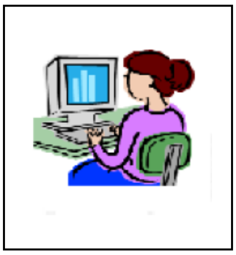

6) Do you have other comments to the speech-language therapy?

____

____

Questionnaire to speech-language pathologist

1) How has it been like to deliver speech-language therapy by video conference?

++	+	0	-	--
Very good	Good	Neither good nor bad	Bad	Very bad

2) Were you satisfied with video quality?

++	+	0	-	--
Very good	Good	Neither good nor bad	Bad	Very bad

3) Were you satisfied with sound quality?

++	+	0	-	--
Very good	Good	Neither good nor bad	Bad	Very bad

4) Did you experience that the participants' language function improved by the speech-language therapy?

++	+	0	-	--
Very good	Good	Neither good nor bad	Bad	Very bad

5) Overall, how satisfied are you with the language therapy that was delivered?

++	+	0	-	--
Very good	Good	Neither good nor bad	Bad	Very bad

6) Do you have other comments to the speech-language therapy?

____

____

____
